# IL-9 Deficiency Promotes Pulmonary Th17 Response in Murine Model of *Pneumocystis* Infection

**DOI:** 10.3389/fimmu.2018.01118

**Published:** 2018-05-25

**Authors:** Ting Li, Heng-Mo Rong, Chao Zhang, Kan Zhai, Zhao-Hui Tong

**Affiliations:** Department of Respiratory and Critical Care Medicine, Beijing Institute of Respiratory Medicine and Beijing Chao-Yang Hospital, Capital Medical University, Beijing, China

**Keywords:** *Pneumocystis* pneumonia, IL-9, Th17 cells, IL-23, alveolar macrophages

## Abstract

**Introduction:**

*Pneumocystis* pneumonia (PCP) remains a severe complication with high mortality in immunocompromised patients. It has been well accepted that CD4^+^ T cells play a major role in controlling *Pneumocystis* infection. Th9 cells were the main source of IL-9 with multifaced roles depending on specific diseases. It is unclear whether IL-9/Th9 contributes to the immune response against PCP. The current study aims to explore the role of IL-9 and the effect of IL-9 on Th17 cells in murine model of PCP.

**Materials and methods:**

Mice were intratracheally injected with 1 × 10^6^ *Pneumocystis* organisms to establish the murine model of *Pneumocystis* infection. *Pneumocystis* burden was detected by TaqMan real-time PCR. Using IL-9-deficient (IL-9^−/−^) mice, flow cytometry, real-time PCR and enzyme-linked immunosorbent assay (ELISA) were conducted to investigate the immune function related to Th17 response in defense against *Pneumocystis* infection.

**Results:**

Reduced *Pneumocystis* burden was observed in lungs in IL-9^−/−^ mice compared with WT mice at 3-week postinfection. IL-9^−/−^mice exhibited stronger Th17 immune responses than WT PCP mice through flow cytometer and real-time PCR. ELISA revealed higher levels of IL-17 and IL-23 in bronchoalveolar lavage fluid from IL-9^−/−^ mice than WT mice. And IL-9 deficiency promoted Th17 differentiation from CD4^+^ naive T cells. IL-17A neutralization increased *Pneumocystis* burden in IL-9^−/−^ mice.

**Conclusion:**

Although similar basic clearance of *Pneumocystis* organisms was achieved in both WT and IL-9^−/−^ PCP mice, IL-9 deficiency could lower *Pneumocystis* organism burden and promote pulmonary Th17 cells response in the early stage of infection.

## Introduction

*Pneumocystis* is an opportunistic fungal pathogen which causes often fatal pneumonia in immunocompromised individuals (1). The incidence and mortality of *Pneumocystis* pneumonia (PCP) in HIV patients have decreased continuously due to highly activate antiretroviral therapy (2); however, PCP rate is increasing in non-HIV compromised patients (3) and the reported mortality of non-HIV PCP patients is as high as 62% in intensive care unit (4).

Host immunity defense mechanisms against *Pneumocystis* organism remains poorly understood. The onset of PCP is typically related to CD4^+^ T cell count less than 200 cells/μL (5), which indicates the key role of this lymphocyte subset in defense against *Pneumocystis* infection. In murine models, depletion of CD4^+^ T cells makes the mice susceptible to *Pneumocystis* infection. When CD4^+^ T cells depletion is stopped and CD4^+^ T cells are transferred into infected mice, the *Pneumocystis* infection is resolved (6). In recent years, studies have down to explore the immune function of specific T helper (Th) subsets during the pathogenesis of PCP, such as IFN-γ-producing CD4^+^ T (Th1) cells, IL-4-producing CD4^+^ T (Th2) cells (7) and CD4^+^CD25^+^FoxP3^+^ regulatory T cells (8, 9).

Th17 cells are clearly distinct from Th1 and Th2 cells and numerous studies have demonstrated that Th17 cells and its signature cytokine IL-17A are important for host defense against various fungal pathogens (10) including *Pneumocystis* (11–14). As reported, IL-17A production during *Pneumocystis* infection is increased (14), and blockage of IL-17 caused a 10,000-fold increase in *Pneumocystis murina* load in the lung of mice compared with wide-type mice (12). Furthermore, Th17 immunity is required for the formation of inducible bronchus-associated lymphoid tissue during *Pneumocystis* infection in mice (13). In addition to the above evidence from animal models, human dendritic cells drive the activation of Th17/IL-17 after stimulated by surface β-glucan components of *Pneumocystis* (11). IL-23 is required for maintaining and extending Th17 cell function in the effector response (15), which is supported by the results that alveolar macrophage (AM) treated with *Pneumocystis* induced increased IL-23 production, and IL-23 was essential for optimal IL-17 production. Moreover, the clearance of *Pneumocystis* was impaired in IL-23-deficient mice (12).

Th9 cells are relatively new subset characterized by production of IL-9 as signature cytokine and this subset can develop from naive T cells stimulated with IL-4 and transforming growth factor β. IL-9 used to be associated with Th2 subset and implicate in the immunity responses of many diseases, including parasitic infection (16), asthma (17) and cancer (18). Previous studies revealed the role of Th2 or IL-9 in fungal infectious diseases. For instance, it is reported that the patients with chronic mucocutaneous candidiasis have a general Th defect including Th2 and Th9 and significant lower production of IL-9 (19). T1/ST2 is a marker for Th2 activation, and T1/ST2 deficiency could result in defective pulmonary fungal control in a murine pulmonary model of cryptococcosis neoformans infection (20). Shellito et al. found that Th2 cells, recruited from draining lymph nodes into lung tissues, were involved in the defense against *Pneumocystis* organisms, furthermore, Th2 response was greater both in lymph nodes and in lung than Th1. Given that IL-9 pleiotropic roles have been extensively described in both inflammation and immunosuppression (21–23), Th9 cell function cannot be segregated as neatly as some other Th subsets. Different from Th2 cells, no studies demonstrate whether Th9 or IL-9 plays a role in the process of *Pneumocystis* infection till now.

Besides Th9, Th17 cells are reported to be capable of producing IL-9 (24). Hence, it is necessary to elaborate the role of Th9/IL-9 on Th17 cell immunity and the relationship between Th17/IL-17 and Th9/IL-9 in different diseases models. The murine model of experimental autoimmune encephalomyelitis was used to explore the influence of IL-9 blockage on Th17-related inflammation response, suggesting IL-9 might play the role as a Th17-mediated cytokine (25). In a murine model of malignant pleural effusion, IL-17 deficiency was observed to inhibit Th9 cell differentiation probably *via* suppressing interferon regulatory factor 4 (IRF4) expression (26). Neutralization of IL-9 reduces Th17 responses in allergic rhinitis, leading to broad anti-inflammatory effects (27). In a population of 41 oral lichen planus patients, positive correlations were found between Th9 and Th17 cells in reticular and erosive oral lichen planus (28). However, no defined conclusion was drawn about the exact relationship between them.

Previous studies have shown that Th17 cells are involved in the immunity responses against *Pneumocystis*, however, no reports have focused on the role of Th9/IL-9 or whether IL-9 could affect Th17 response in PCP. In the present study, we provided insights into the function of IL-9 during clearance of *Pneumocystis* and explore the effect of IL-9 on Th17 responses in *Pneumocystis* infection using the IL-9 deficient (IL-9^−/−^) mice.

## Materials and Methods

### Animals

Healthy male BALB/c and severe combined immunodeficiency (Scid) mice were purchased at 6–7 weeks of age from Vital River Lab Animal Co., Ltd. (Beijing, China), weighting 20–22 g. IL-9^−/−^ mice (BALB/c background) were kindly provided by Professor Andrew McKenzie of MRC Laboratory of Molecular Biology, Cambridge, UK (29). IL-17-deficient (IL-17^−/−^) mice (C57BL/6 background) were generated as previously described and provided by Dr. Iwakura of University of Tokyo (30). Animals were housed in specific pathogen-free conditions. All of the procedures were approved by the Capital Medical University Animal Care and Use Committee.

### Murine Model of *Pneumocystis* Infection

*Pneumocystis murina* organisms (ATCC, Manassas, VA, USA) was maintained in Scid mice, and the infected lungs were removed and homogenized to get *Pneumocystis* organisms for inoculation as described earlier (12). Mice were intratracheally injected with 1 × 10^6^ *Pneumocystis* organisms diluted in 100 μL PBS, while non-infection mice were inoculated with lung homogenates from uninfected Scid mice. Mice were sacrificed at serial time intervals under anesthesia. *Pneumocystis* infection was confirmed in lung homogenates stained with Diff-Quick (Baso Diagnostics Inc., Zhuhai, China), meanwhile, secondary infections were excluded. *Pneumocystis* quantification was performed by TaqMan quantitative PCR as previously described (31) using the right inferior mouse lungs (see below). The left lungs were harvested and minced into pieces, then digested and filtered to get isolated leukocytes (14) for further flow cytometry analysis and cell counting.

### TaqMan Real-Time PCR for *Pneumocystis* Quantification

A previously described TaqMan PCR method with minor modifications was used to quantify *Pneumocystis* lung burden (31). Briefly, a portion of *Pneumocystis* muris rRNA (GenBank accession no. AF257179) was amplified and then quantitated as standard sample for assay. The TaqMan PCR primers for mouse *Pneumocystis* rRNA were 5′-AGGTGAAAAGTCGAAAGGGAAAC-3′ and 5′-AAAACCTCTTTTCTTTCACTCAGTAACA-3′. The probe sequence was 5′-FAM-CCCAGAATAATGAATAAAG-MGBNFQ-3′. The real-time PCR was performed using one-step Probe RT-PCR kit (Takara Bio, Dalian, China) on the ABI Prism 7500 Sequence Detection System instrument. A standard curve was generated by amplifying known copy number of *Pneumocystis* rRNA template in five serial of 1:5 dilutions per PCR reaction, and data from infected mouse lung were converted to rRNA copy numbers according to the stand curve to reflect the *Pneumocystis* burden.

### Bronchoalveolar Lavage

After anesthetized with pentobarbital, mice were sacrificed by collecting blood from eyeballs. The trachea was cannulated with a polyethylene 18 G catheter. Lungs underwent lavage three times with 800 μL PBS to get bronchoalveolar lavage fluid (BALF). BALF cells were collected by centrifugation at 400 *g* for 10 min and resuspended in PBS for flow cytometry or in medium for further culture. The supernatant was stored at −80°C for the cytokine measurement.

### Flow Cytometry

All of the different fluorochrome-conjugated monoclonal antibodies (mAbs), including anti-CD45, -CD3, -CD4, -CD8, -F4/80, -CD11c, -CD11b, -Ly-6G, -IL-23 receptor (IL-23R), -IFN-γ, -IL-4 and -IL-17A, were purchased from BD Pharmingen (San Diego, CA, USA) or eBioscience (San Diego, CA, USA).

For intracellular detection of cytokines, cells were stimulated for 4 h at 37°C, added with 50 ng/mL phorbol myristate acetate (Sigma-Aldrich, St. Louis, MO, USA) and 1 µg/mL ionomycin (Sigma-Aldrich, St. Louis, MO, USA) in the presence of Brefeldin A (10 µg/mL, Enzo Life Science). Cells were surface-stained with extracellular mAbs in PBS + 2% heat-inactivated fetal bovine serum (FBS, Gibco) for 20 min at 4°C. Cells were resuspended in a fixation/permeabilization solution (Cytofix/Cytoperm; BD Pharmingen) and incubated with intracellular cytokine mAbs for 30 min at 4°C. Cells were then washed with permeabilization buffer and then resuspended in PBS + 2% FBS for flow cytometric analysis (FACS Canto II; BD Biosciences, San Jose, CA, USA). The absolute cell counting was determined with BD Trucount Tubes (BD Biosciences, San Jose, CA, USA).

### Enzyme-Linked Immunosorbent Assay (ELISA)

Mouse IL-17A and IL-23p19 were detected by the ELISA kits according to manufacturer’s protocols. The ELISA kits are from eBioscience (San Diego, CA, USA).

### Real-Time PCR

RNA was extracted from homogenized tissue and cells using TRIzol reagent (Invitrogen Life Technologies, Carlsbad, CA, USA) and reverse transcribed according to the PrimeScript TM II 1st Strand cDNA Synthesis Kit (Takara Bio, Dalian, China). cDNA were used as templates in real time PCR with the SYBR Premix Ex Taq TM II ROX plus (Takara Bio, Dalian, China) using the ABI Prism 7500 Sequence Detection System instrument. Target gene expression was calculated using 2^−ΔΔCt^ method after normalization to GAPDH gene expression. Primers sequences used for PCR are listed in Table S1 in Supplementary Material.

### Differentiation of Th17 Cells *In Vitro*

Mononuclear cells from the spleens of WT and IL-9^−/−^ mice were isolated by Ficoll-Hypaque gradient centrifugation. Naive CD4^+^CD62L^+^ T cells were isolated by CD4^+^CD62L^+^ T cell isolation kit II (Miltenyi Biotec, Aubum, CA, USA) according to the manufacturer. The purity of naive CD4^+^CD62L^+^ T cells was above 90%, as measured by flow cytometry. Purified naive CD4^+^ T cells were cultured in RPMI-1640 medium containing 10% FBS in the presence of plate-bound anti-CD3 (10 µg/mL) and anti-CD28 (2 µg/mL) mAbs. Cytokines and mAbs used for Th17 cell differentiation were TGF-β (5 ng/mL), IL-6 (20 ng/mL), IL-23 (50 ng/mL), anti-IFN-γ mAb (10 µg/mL), and anti-IL-4 mAb (10 µg/mL) (32). After 3, 5 and 7 days of culture, cells were restimulated for 4 h with phorbol myristate acetate and ionomycin in the presence of Brefeldin A before intracellular staining of cytokines as described in Section “Flow Cytometry.” The supernatant from differentiated cells was collected and stored at −80°C for further analysis.

### Apoptosis Analysis *In Vitro*

The AMs were isolated from BALF of IL-17^−/−^ mice through removal of non-adherence cells after cultured at 37°C, 5% CO_2_ for 2 h. The purity of the isolated AMs was above 95% measured by flow cytometry. Murine recombinant IL-17A (0, 100, and 200 ng/mL) from PeproTech (Rocky Hill, NJ, USA) was used to treated AMs for 48 h, then AMs were collected with tryptic digestion and washed by cold PBS and subjected to a PI/Annexin V-FITC Apoptosis Detection Kit (BD). The apoptosis analysis was analyzed by FACS Canto II (BD Biosciences, San Jose, CA, USA) within 30 min.

### IL-17A Neutralization *In Vivo*

IL-17A neutralization was conducted as described previously (33). Briefly, mice were inoculated intraperitoneally with 500 µg of anti-mouse IL-17A clone 17F3 (BioXcell, West Lebanon, NH, USA) or isotype control in 100 µL of PBS the day before infection and twice a week thereafter until experiment completion.

### Statistical Analysis

Data are shown as mean ± SEM. Statistical analyses were performed by Prism 5.0 (Graphpad Software). Two-tailed Student’s *t*-test was used for experiments with two groups. When three groups were compared, one-way ANOVA analysis with Bonferroni posttest was applied. For analysis of *Pneumocystis* burden on multiple time points in the lungs of infected mice, two-way ANOVA followed by a Bonferroni multiple comparison procedure was performed. *P* value less than 0.05 was considered to indicate statistical significance.

## Results

### IL-9 Deficiency Reduced the *Pneumocystis* Burden

**Figure 1:**
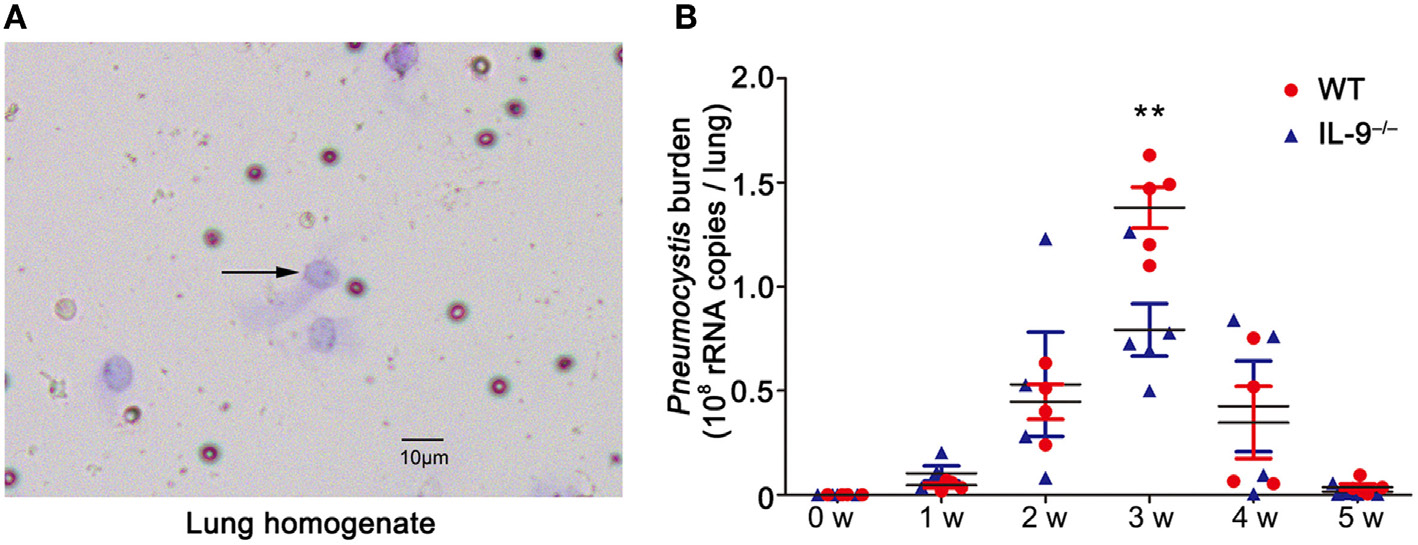
IL-9 deficiency reduced the *Pneumocystis* burden. The mice were infected with 10^6^ *Pneumocystis* organisms and were sacrificed at weeks 1, 2, 3, 4, and 5. **(A)** The lung from mice was homogenized and smeared quickly for Diff-Quick stain. The black arrow indicates *Pneumocystis* murina asci (200×). **(B)** The *Pneumocystis* burden was detected by TaqMan quantitative PCR. The uninfected mice had no detectable *Pneumocystis* burden. The results were from three experiments using three to five mice per group per time point. **P < 0.01 compared to WT group at the same time point by a two-way ANOVA followed by a Bonferroni multiple comparison procedure.

WT and IL-9^−/−^ mice were intratracheally injected with 10^6^ *Pneumocystis* murina organisms and sacrificed at a series of postinfection time-points. The Diff-Quick stain of lung homogenate was performed on every mouse to provide the microbiology evidence of *Pneumocystis* organisms, confirming the establishment of *Pneumocystis* infection model (Figure 1A). The *Pneumocystis* burden peaked at 3-week postinfection and successful clearance of *Pneumocystis* was basically achieved in both groups at 5-week postinfection. Though IL-9 deficiency seemed to had no significant effect on the final clearance of *Pneumocystis* organisms, IL-9^−/−^ mice demonstrated a significantly decreased *Pneumocystis* burden compared to WT mice [(1.39 ± 0.10) × 10^8^ vs. (7.91 ± 1.26) × 10^7^ copies/lung, *P* < 0.01] (Figure 1B) at 3-week postinfection, suggesting that IL-9 deficiency might contribute to fighting against *Pneumocystis* infection.

### IL-9 Deficiency Promoted Th17 Responses During *Pneumocystis* Infection

**Figure 2:**
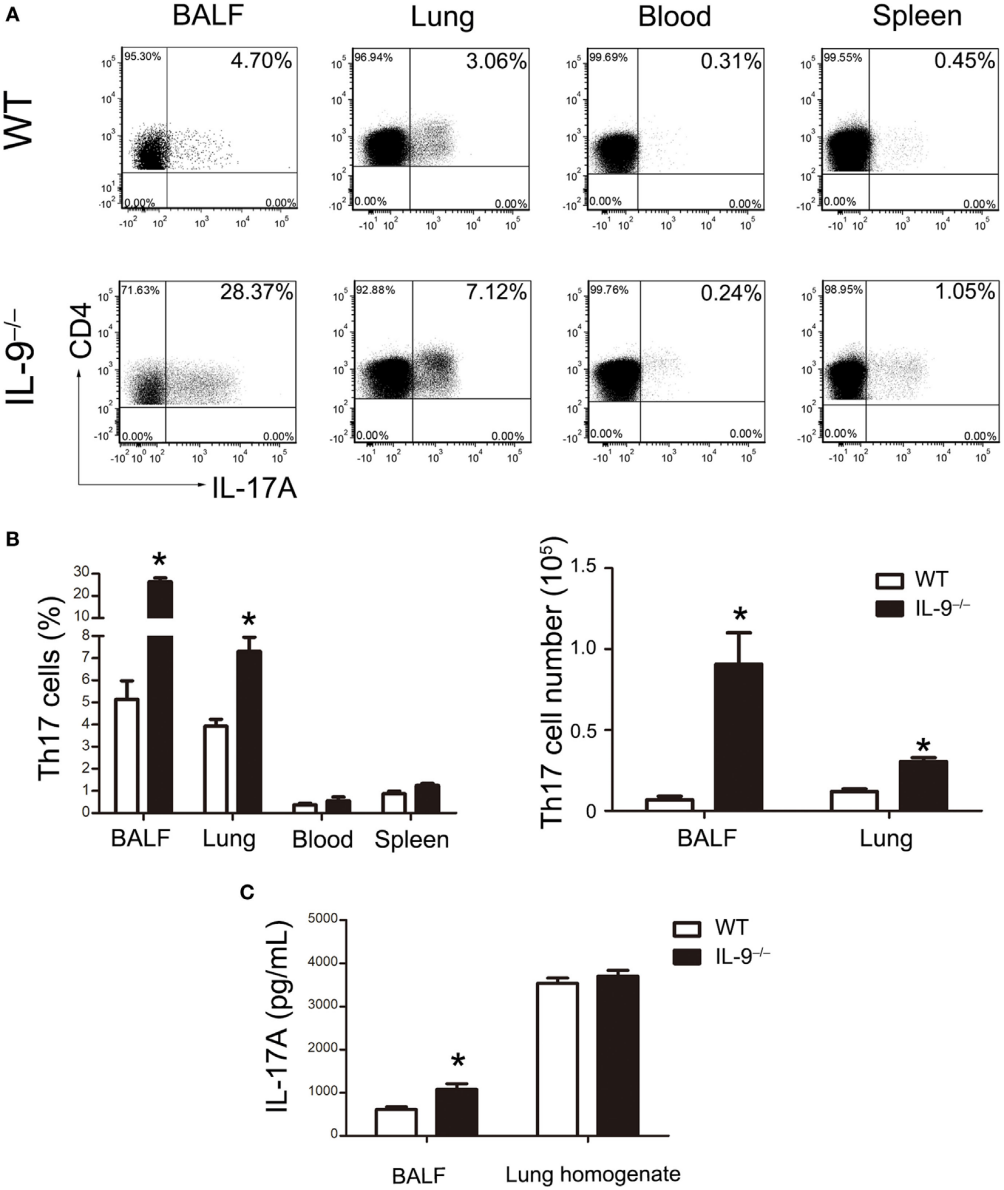
IL-9 deficiency promoted Th17 responses during *Pneumocystis* infection. **(A)** Cells were isolated from bronchoalveolar lavage fluid (BALF), lung, spleen, and blood and then stimulated as described previously. The representative flow cytometric dot plots of Th17 cells (CD4^+^IL-17A^+^, gated on cells from CD3^+^CD4^+^ lymphocytes) from WT mice (top panel) and IL-9^−/−^ mice (bottom panel) 3-week post-*Pneumocystis* infection. **(B)** The comparisons of Th17 percentage (left) and absolute number (right) were shown from different tissues in WT and IL-9^−/−^ PCP mice. Data were presented as mean ± SEM from three to five mice per group. **(C)** The IL-17A levels were determined in BALF and lung homogenate supernatants by ELISA from WT and IL-9^−/−^ PCP mice at 3-week postinfection. The IL-17A concentrations were expressed as mean ± SEM from four to five mice per group. The results were from three independent experiments. *P < 0.05 compared to WT group by Student’s *t*-test.

In order to investigate the mechanism leading to the difference of *Pneumocystis* burden between IL-9^−/−^ and WT mice, CD4^+^ Th cells subsets including Th1, Th2 and Th17 cells were measured by flow cytometry in BALF, lung, blood and spleen. No significant difference was observed in the percentage of Th1 or Th2 cells between the two groups (Figure S1 in Supplementary Material). Interestingly, the percentage of Th17 cells in CD4^+^ lymphocytes was significantly higher in the IL-9^−/−^ mice than WT mice in the BALF [(5.14 ± 0.83 vs. 26.42 ± 1.71)%, *P* < 0.001] and lungs [(3.93 ± 0.31 vs. 7.31 ± 0.64)%, *P* = 0.003] at 3-week postinfection, when *Pneumocystis* burden peaked during infection. And the absolute numbers of Th17 cells were higher in BALF [(6.93 ± 2.05) × 10^3^ vs. (9.07 ± 1.93) × 10^4^ cells/mice, *P* = 0.005] and lung [(1.20 ± 0.16) × 10^4^ vs. (3.06 ± 0.23) × 10^4^ cells/mice, *P* < 0.001] from IL-9^−/−^ mice than those from WT mice (Figures 2A,B). A significant increase on IL-17A concentration was observed in BALF of IL-9^−/−^ mice than WT mice [(610.0 ± 57.1 vs. 1076.0 ± 132.2) pg/mL, *P* = 0.015] (Figure 2C). To our disappointment, no significant difference was found in lung homogenate IL-17A concentration between WT and IL-9^−/−^ PCP mice. Thus, these data showed that pulmonary Th17 response was enhanced in IL-9^−/−^ mice during *Pneumocystis* infection.

### IL-9 Deficiency Enhanced Th17 Cells Differentiation *In Vitro*

**Figure 3:**
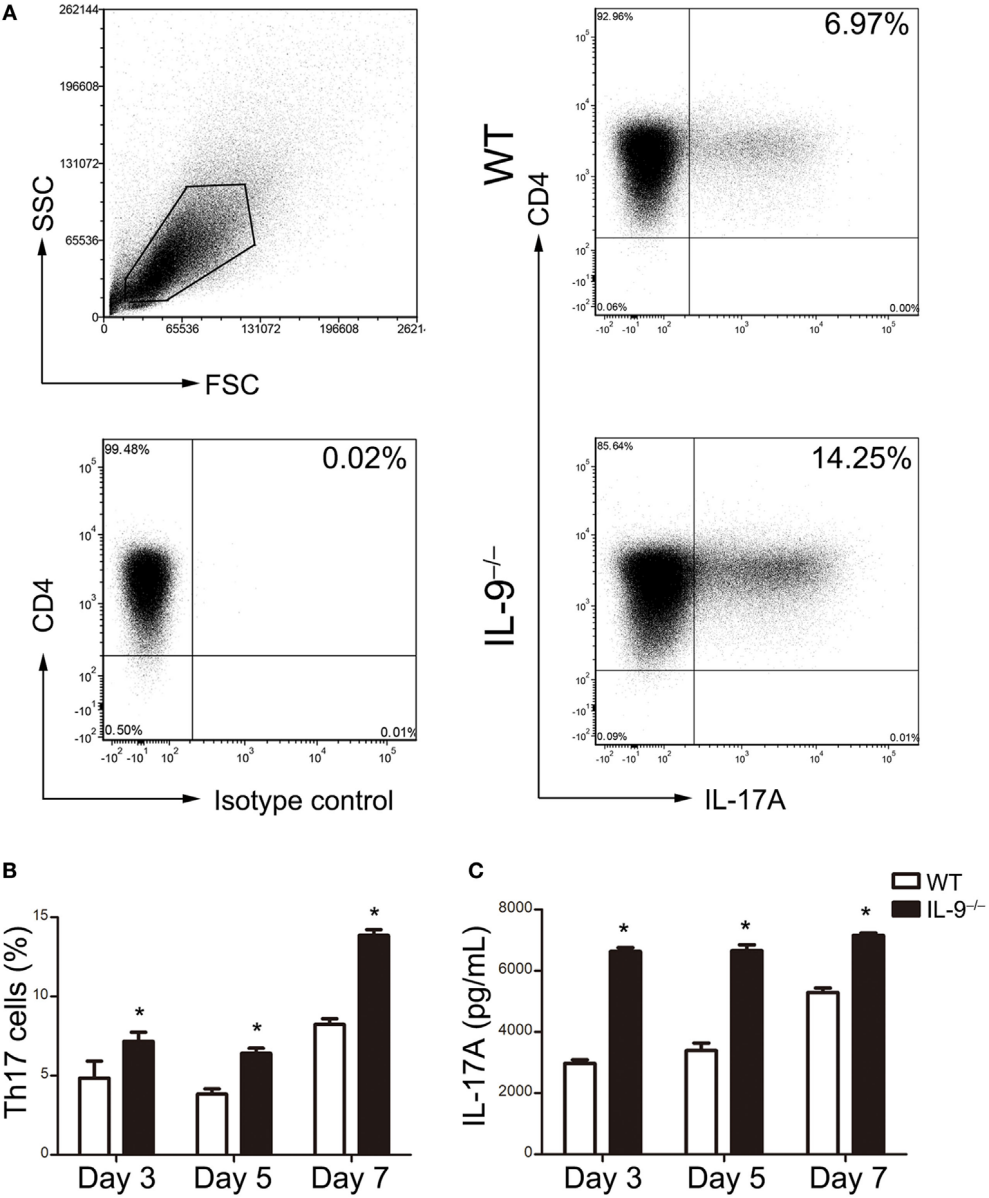
IL-9 deficiency enhanced T-helper (Th)17 cells differentiation *in vitro*. Naive CD4^+^ CD62L^+^ T cells were purified from splenocytes of 8-week-old WT and IL-9^−/−^ mice and cultured under Th17 differentiation condition (as described in Section “Materials and Methods”). Cells were obtained on day 3, 5, 7 and assessed for IL-17 expression by flow cytometry. The culture supernatants were measured for IL-17A concentration by ELISA. **(A)** Representative flow cytometric dot plots were presented to show the Th17 cells differentiated from naive CD4^+^ T cells on day 7. Comparisons of Th17 cell percentage **(B)** and IL-17A concentrations **(C)** were demonstrated between WT and IL-9^−/−^ mice. Data were presented as mean ± SEM of three to four samples per group from three independent experiments. *P < 0.05 compared to WT group at corresponding day by Student’s *t*-test.

The naive CD4^+^ T cells were purified from IL-9^−/−^ and WT mice splenocytes and then cultured in the presence of Th17 differentiation condition. Flow cytometric analysis was performed to detect the percentage of Th17 cells on different time points (Figure 3A). The differentiation of Th17 cells from IL-9^−/−^ mice were all significantly increased compared with WT mice after 3, 5 and 7 days culture (all *P* < 0.05, Figure 3B). Similarly, the supernatant of the cultured differentiated cells from IL-9^−/−^ mice presented a higher IL-17A concentration than that from WT mice (all *P* < 0.05, Figure 3C). All the above results suggested that IL-9 deficiency enhanced Th17 cells differentiation.

### Th17/IL-17-Related Genes Expression Was Upregulated in Lungs of IL-9^−/−^ PCP Mice

**Figure 4:**
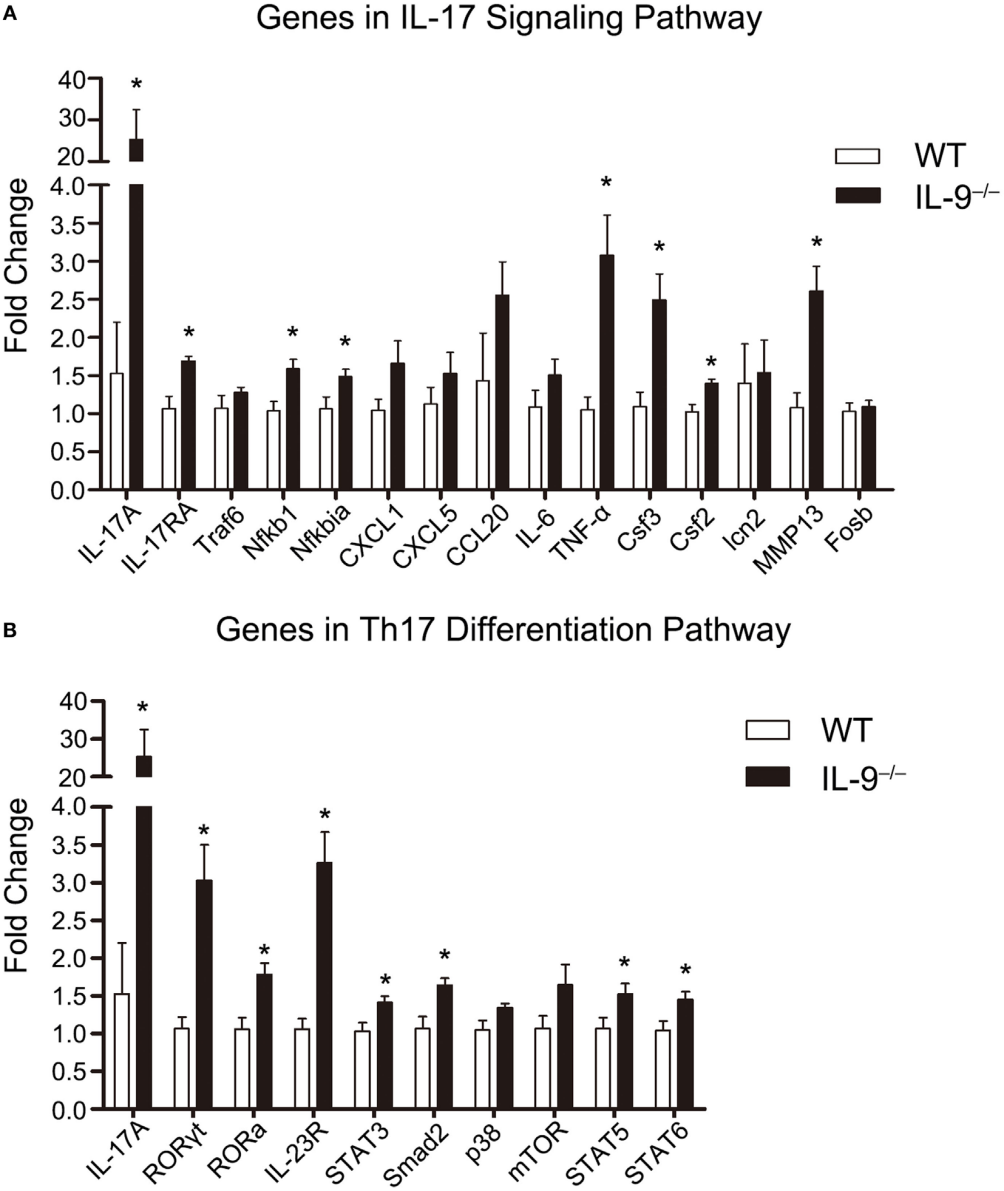
T-helper (Th)17/IL-17-related genes expression was upregulated in lungs of IL-9^−/−^ PCP mice. The whole-lung RNA was extracted from WT and IL-9^−/−^ mice at 3-week postinfection. The genes which are related to IL-17 signaling pathway **(A)** and Th17 differentiation pathway **(B)** were detected through real-time PCR for mRNA expression between the two groups. The fold change was figured out using the 2^−ΔΔCT^ normalized to GAPDH. Reactions were performed in triplicate for *n* = 5–7 samples per group. Data were presented as mean ± SEM and the graphs were representative of three independent experiments. *P < 0.05 compared to WT group by Student’s *t*-test.

The mRNA expression of genes involved in IL-17 signaling pathway, including IL-17A, IL-17RA, Traf6, Nfkb1, Nfkbia, CXCL1, CXCL5, CCL20, IL-6, TNF-α, Csf3, Csf2, lcn2, MMP13, and Fosb, were measured in lungs of both IL-9^−/−^ PCP and WT PCP mice 3-week postinfection, and the results were shown in Figure 4A. The mRNA levels of IL-17A, IL-17RA, Nfkb1, Nfkbia, TNF-α, Csf3, Csf2 and MMP13 were significantly higher in IL-9^−/−^ PCP group than WT PCP group (all *P* < 0.05). The mRNA expression of genes involved in Th17 differentiation pathway, including IL-17A, Rorgt, Rora, IRF4, IL-23R, STAT3, Smad2, p38, mTOR, STAT5 and STAT6, were detected in lungs of both IL-9^−/−^ PCP and WT PCP mice as shown in Figure 4B. It was observed that the mRNA expression of IL-17A, RORγt, RORa, IRF4, IL-23R, STAT3, Smad2, STAT5 and STAT6 were significantly increased in IL-9^−/−^ PCP mice compared with WT PCP mice (all *P* < 0.05).

### IL-23 Was Higher in BALF From IL-9^−/−^ PCP Mice

**Figure 5:**
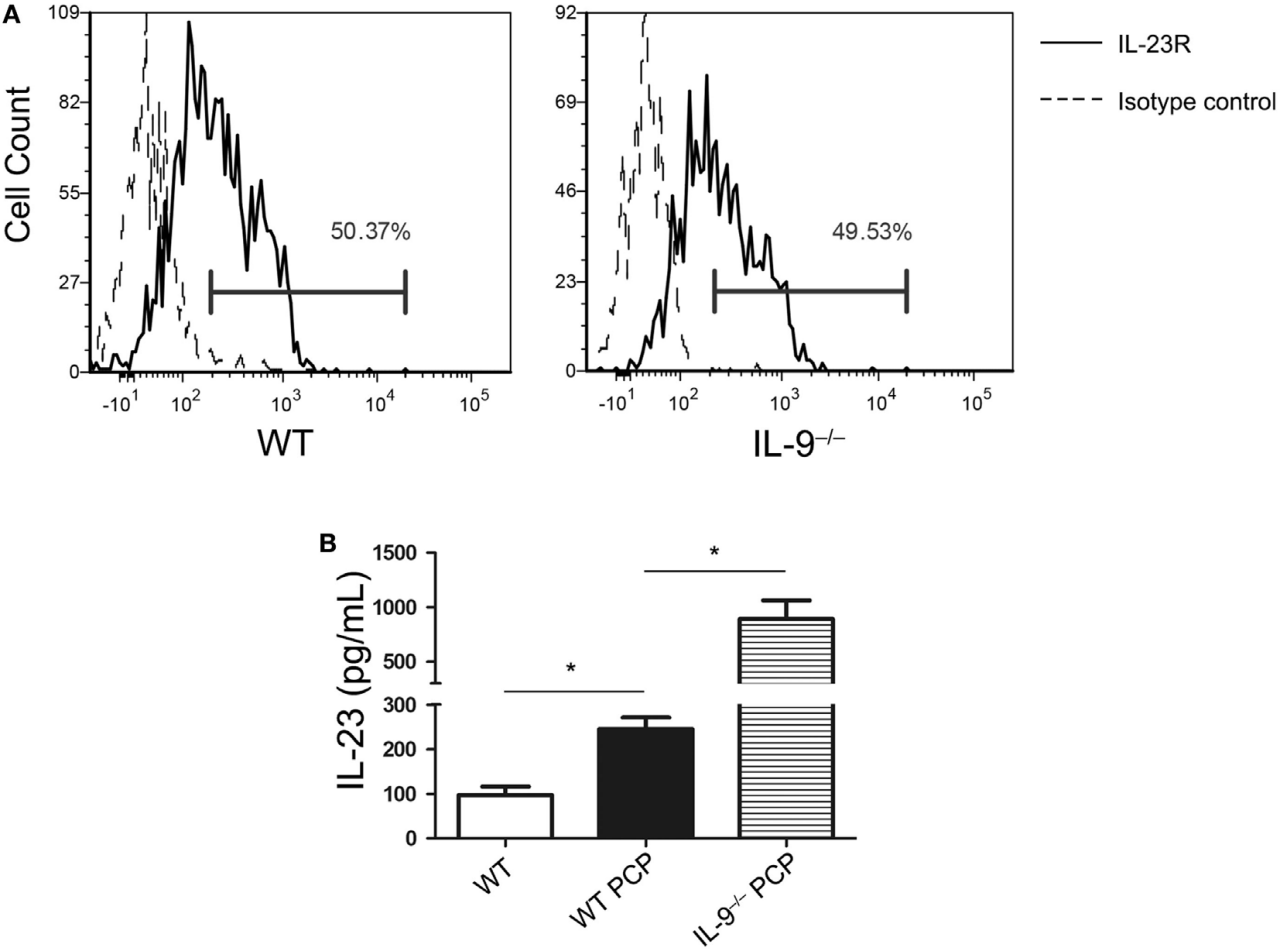
IL-23 was higher in bronchoalveolar lavage fluid (BALF) from IL-9^−/−^ *Pneumocystis* pneumonia (PCP) mice. **(A)** Cells from BALF were detected for IL-23R expression on T-helper (Th) 17 cells through Flow cytometry. About 50% of Th17 cells isolated from WT (left) and IL-9^−/−^ (right) PCP mice expressed IL-23R (solid line histogram) according to isotype control (dashed line histogram). **(B)** BALF was centrifuged from uninfected WT mice, WT PCP mice and IL-9^−/−^ PCP mice, and then the supernatant was examined for IL-23. Data represented mean ± SEM (*n* = 4–6 per group) from three independent experiments. *P < 0.05 compared with WT PCP group by Student’s *t*-test.

It is widely reported that IL-23 could promote Th17 cells expansion or survival, suggesting the close relationship between IL-23 and Th17/IL-17 in immune response (34). It should be noted that higher Th17 cells were observed in BALF than lung tissue in both IL-9^−/−^ and WT PCP mice, and IL-9^−/−^ mice showed a more obvious gap between the BALF and lung tissue. In order to investigate the mechanisms how Th17 cells were drawn into the alveolar spaces, the supernatant of BALF were detected for the concentration of cytokines CCL20 and IL-23. We found the concentration of CCL20 from BALF between IL-9^−/−^ PCP and WT PCP mice were similar (Figure S2 in Supplementary Material). The majority of Th17 cells in BALF obtained from IL-9^−/−^ or WT PCP mice expressed IL-23 receptor, which presented about 50% of the Th17 population, with no significant difference between the two groups (Figure 5A). IL-23 level was much higher in the BALF from *Pneumocystis*-infected WT mice as compared to that from uninfected mice [(96.91 ± 19.0 vs. 245.1 ± 26.3) pg/mL, *P* = 0.003]. In the postinfection mice, production of IL-23 in BALF from IL-9^−/−^ mice was higher than that from WT mice [(245.1 ± 26.3 vs. 892.3 ± 170.1) pg/mL, *P* = 0.003] (Figure 5B).

### IL-17 Inhibited AMs Apoptosis *In Vitro*

**Figure 6:**
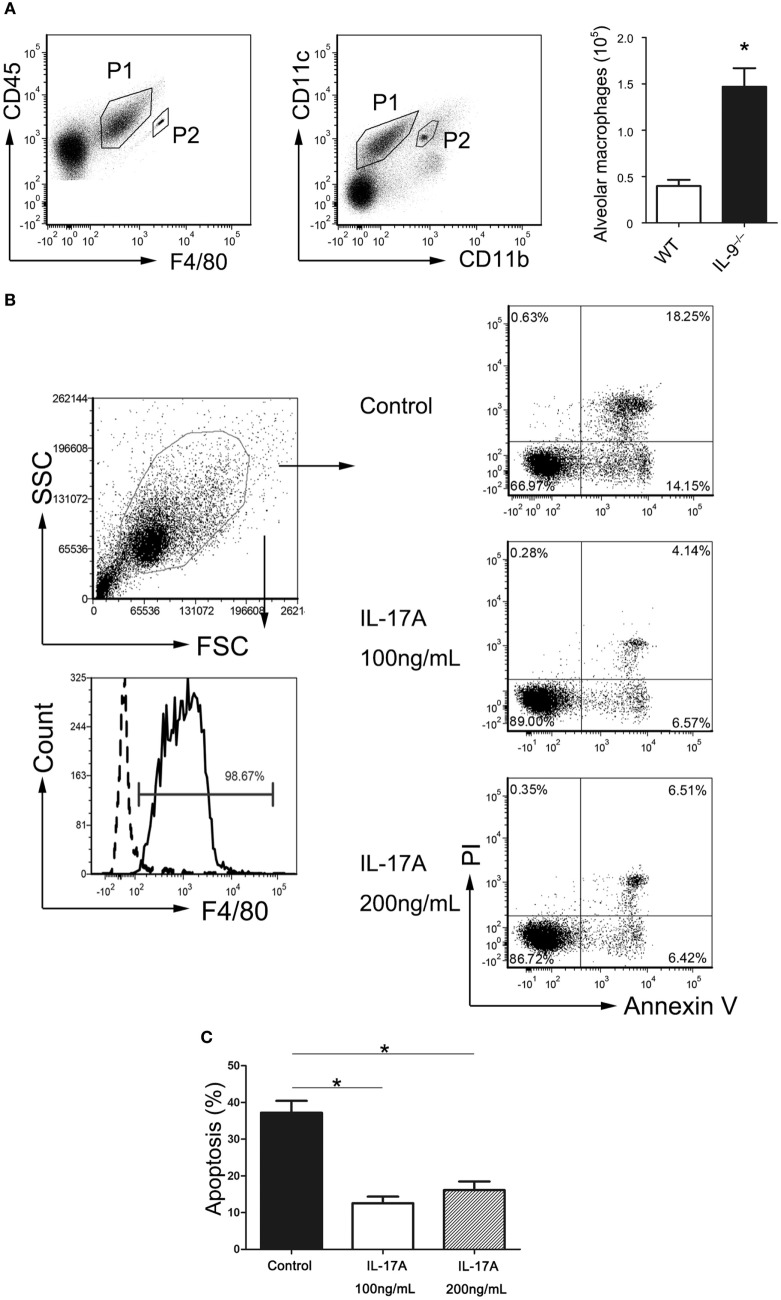
IL-17 inhibited alveolar macrophages apoptosis. **(A)** Representative flow cytometric dot plots showed that alveolar macrophages (P1) were gated as CD45^+^F4/80^+^CD11b^int^CD11c^+^ cells from bronchoalveolar lavage fluid (BALF), and beads (P2) were used for absolute number counting. The comparison of alveolar macrophage absolute number was presented from BALF of IL-9^−/−^ mice and WT mice 3-week post-*Pneumocystis* infection. Data were from four to five samples per group from three independent experiments. *P < 0.05 compared to WT group by Student’s *t*-test. **(B)** The alveolar macrophages (CD45^+^F4/80^+^) were isolated from IL-17^−/−^ mice BALF, and the purity was about 98% (left panel). Determination of the proportion of apoptotic cells contained cells in both early stage (Annexin V^+^/PI^−^) and late stage (Annexin V^+^/PI^+^) apoptosis (right panel). **(C)** Alveolar macrophages cultured with added rmIL-17A (100 or 200 ng/mL) demonstrated significantly reduced apoptosis proportion compared to control group without rmIL-17A. Data were from four to five samples per group from three independent experiments. *P < 0.05 compared to control group by Student’s *t*-test.

It is widely reported that phagocytosis by AMs is the predominant mechanism of *Pneumocystis* clearance from the lungs (35). Our previous data suggested that IL-9^−/−^ mice recruited more AMs (CD45^+^F4/80^+^CD11b^int^CD11c^+^) during *Pneumocystis* infection (Figure 6A). To determine if the elevated IL-17 level in IL-9^−/−^ mice BALF affects the AMs, the AMs were isolated from the uninfected IL-17^−/−^ mice and cultured in different conditions till apoptosis analysis by flow cytometry. Cells exposed to murine recombinant IL-17A at both 100 and 200 ng/mL exhibited significantly decreased apoptosis when compared with control cells in absence of IL-17A [(37.2 ± 3.2 vs. 12.5 ± 1.8)%, *P* < 0.001; (37.2 ± 3.2 vs. 16.1 ± 2.4)%, *P* = 0.003] (Figures 6B,C), which indicated that IL-17 might protect AMs from apoptosis and then contribute to the clearance of *Pneumocystis* with increased AM number. There was no distinguished difference in AM apoptosis proportion between the two groups at different IL-17A concentrations. However, whether or not IL-17 influences the AMs apoptosis during *Pneumocystis* infection *in vivo* is worthy of further investigation.

### IL-9^−/−^ Mice Recruited More Neutrophils During *Pneumocystis* Infection

**Figure 7:**
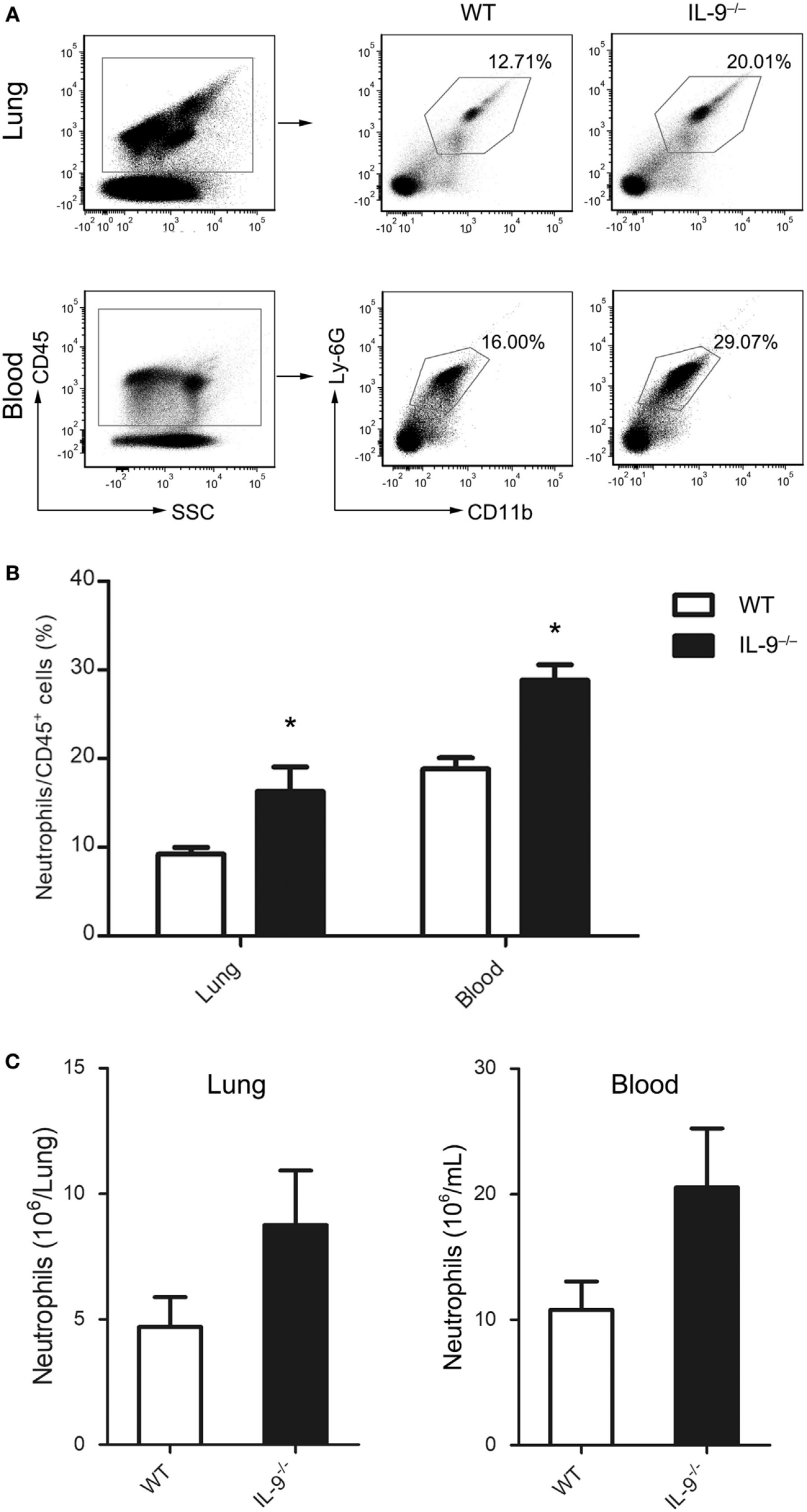
IL-9^−/−^ mice recruited more neutrophils during *Pneumocystis* infection. Neutrophils from blood and lung were determined by flow cytometry in WT and IL-9^−/−^ mice at 3-week after *Pneumocystis* infection. **(A)** The percentage of neutrophils (CD45^+^CD11b^+^Ly-6G^+^) in all CD45^+^ cells were shown in the representative flow cytometric dot plots of three independent experiments. The percentage **(B)** and absolute numbers **(C)** were higher in IL-9^−/−^ mice than WT mice. Data are shown as mean ± SEM (*n* = 4–5 per group). *P < 0.05 compared to WT group by Student’s *t*-test.

Known as an important factor for neutrophil recruitment and activation (36), IL-17A plays a role in defense against various pulmonary infections (37). The difference of Th17 cells between both groups prompted a further analysis of neutrophils infiltration in lungs and blood of IL-9^−/−^ and WT PCP mice. After 3-week postinfection, mice of IL-9^−/−^ and WT groups were sacrificed. The percentage and number of neutrophils (CD45^+^CD11b^+^Ly-6G^+^) were measured by flow cytometer (Figure 7A). Compared with WT PCP mice, the neutrophil percentages were higher in lung [(16.3 ± 2.7 vs. 9.2 ± 0.8)%, *P* = 0.038] and blood [(28.8 ± 1.8 vs. 18.8 ± 1.3)%, *P* = 0.004] of IL-9^−/−^ PCP mice (Figure 7B). And neutrophil absolute numbers were higher in both lung [(8.76 ± 2.16) × 10^6^ vs. (4.69 ± 1.19) × 10^6^ cells/mice, *P* = 0.138] and blood [(20.55 ± 4.69) × 10^6^ vs. (10.78 ± 2.26) × 10^6^ cells/mL, *P* = 0.110] of *Pneumocystis*-infected IL-9^−/−^ mice compared with WT mice, with no significant difference (Figure 7C).

### IL-17A Contributed to the Lower *Pneumocystis* Burden in IL-9^−/−^ Infected Mice

**Figure 8:**
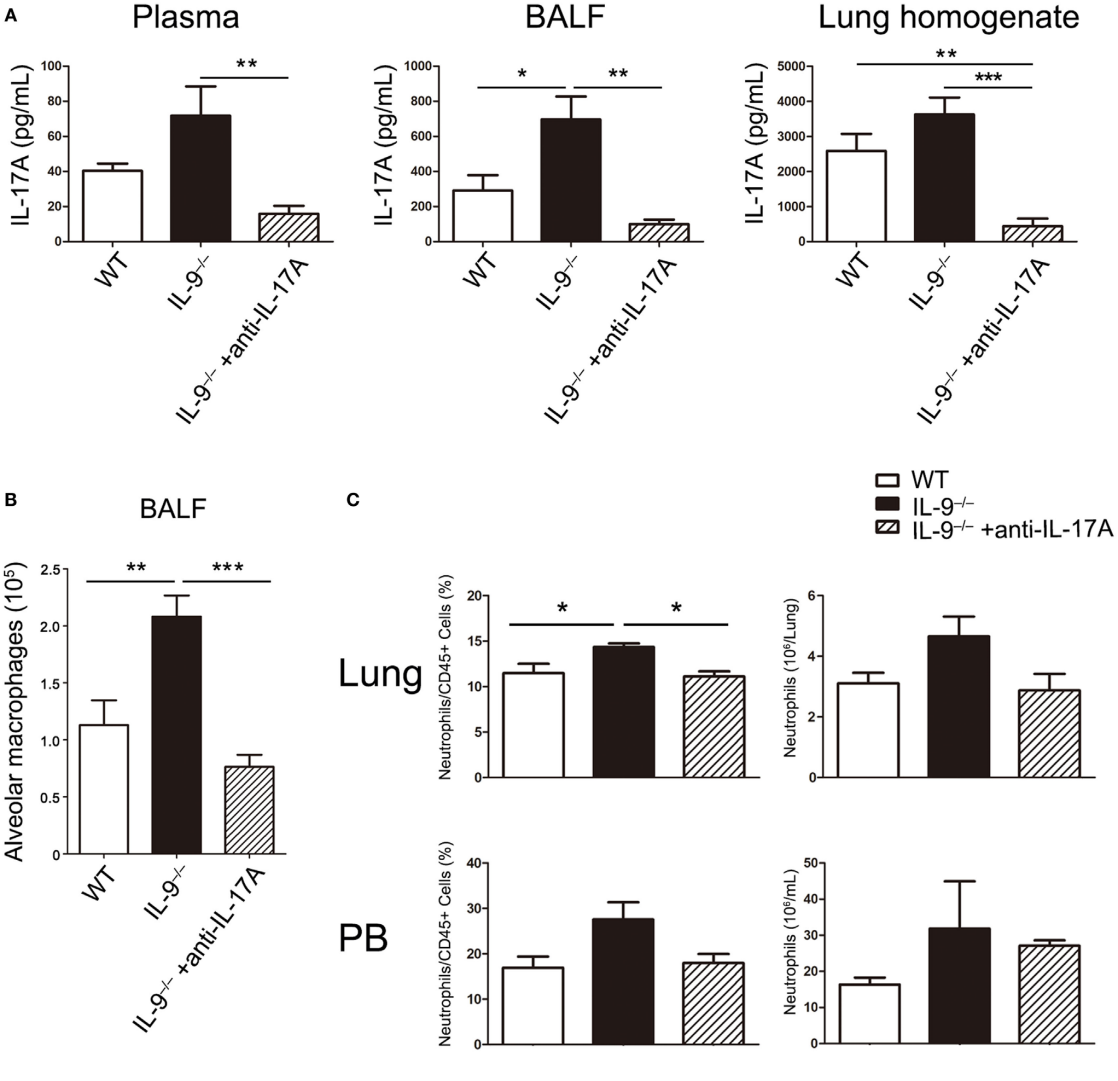

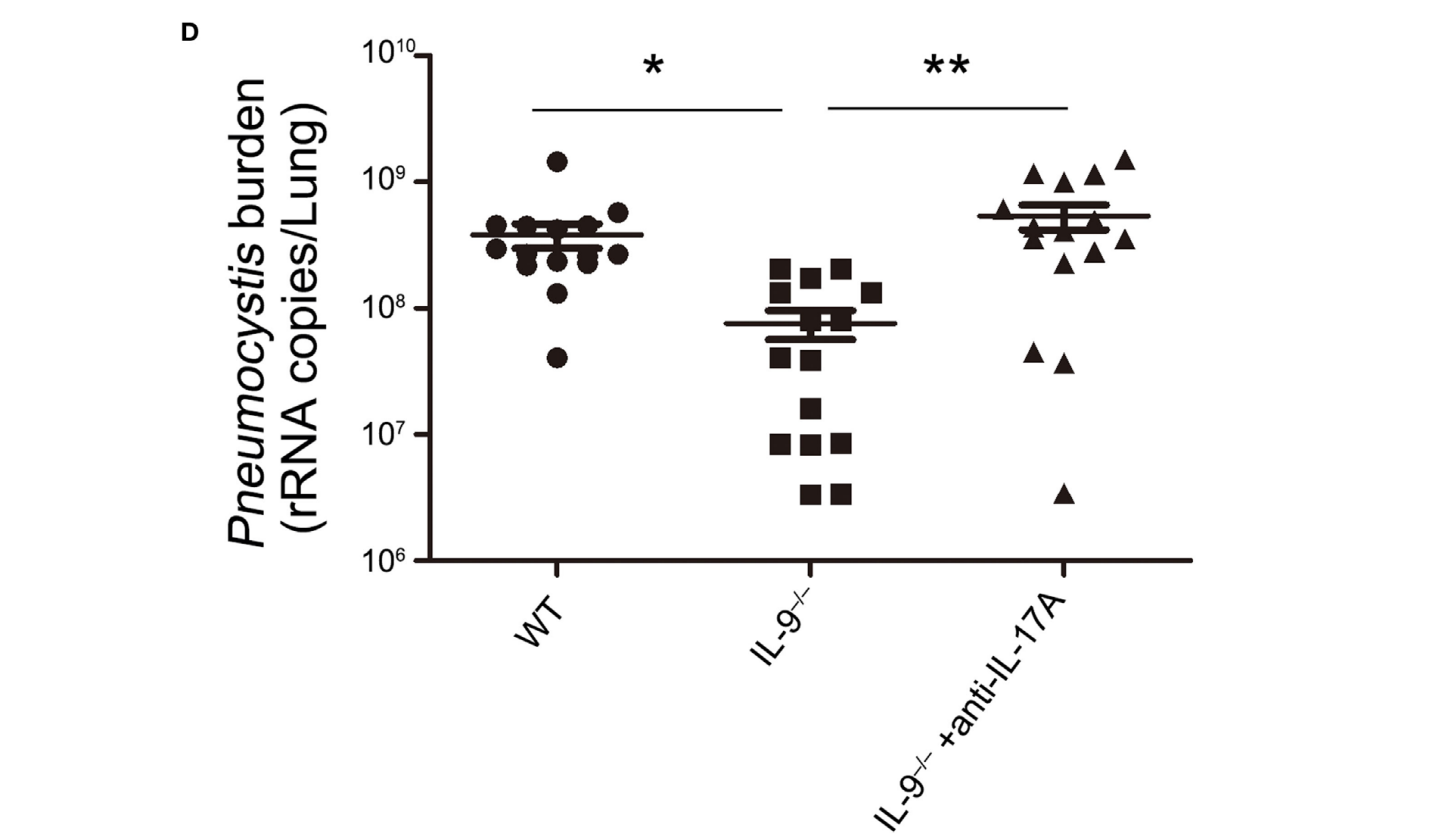
IL-17A neutralization *in vivo* impaired the *Pneumocystis* clearance in IL-9^−/−^ mice. One group of IL-9^−/−^ mice received anti-IL-17A monoclonal antibody as in Section “Materials and Methods” (IL-9^−/−^ + anti-IL-17A). At the same time, another group of IL-9^−/−^ mice (IL-9^−/−^) and one group of WT mice (WT) received isotype control. All of the above three groups were intratracheally injected with *Pneumocystis* organisms and sacrificed at 3-week postinfection. **(A)** The IL-17A levels were determined in plasma, bronchoalveolar lavage fluid (BALF) and lung homogenate supernatants by ELISA from the three *Pneumocystis*-infected mouse groups. **(B)** The comparison of alveolar macrophage (defined as CD45^+^F4/80^+^CD11b^int^CD11c^+^) absolute number was presented from BALF of the three groups mice measured by flow cytometry. **(C)** The comparisons of neutrophil (defined as CD45^+^CD11b^+^Ly-6G^+^) percentage and absolute number in lung (top panel) and blood (bottom panel) were shown from the three groups of mice, which were detected by flow cytometry. Data in the graphs were represented as mean ± SEM (*n* = 6 per group) and were representative of three independent experiments. **(D)** The *Pneumocystis* burden was detected by TaqMan quantitative PCR, and expressed by the copies of *Pneumocystis* rRNA per lung from the three groups. Data in the graph were represented as mean ± SEM of 15 mice per group from three independent experiments. One-way ANOVA test with Bonferroni postanalysis was performed to calculate statistical significance. *P < 0.05, **P < 0.01, and ***P < 0.001.

The results indicated that IL-9^−/−^ mice demonstrated higher Th17 responses and lower *Pneumocystis* burden at 3-week postinfection compared with WT mice. Although previous studies have showed that Th17 cells were involved in the immunity responses against *Pneumocystis* infection and blockage of IL-17A caused higher *Pneumocystis* burden in WT mice. To study the association between the higher Th17 cell response and lower *Pneumocystis* burden, we neutralized IL-17A in *Pneumocystis*-infected IL-9^−/−^ mice and injected isotype antibodies to WT and IL-9^−/−^ mice as control. At 3-week postinfection, mice from the three groups were sacrificed. IL-17A neutralization was confirmed by the significant cytokine reduction in the plasma, BALF and lung homogenate as shown in Figure 8A. Furthermore, the previously observed phenotypes in IL-9^−/−^ mice compared with WT mice, including elevated number of AMs in BALF and percentage of neutrophils in blood and lung, were reverted while receiving IL-17A neutralization (Figures 8B,C). And more notably, IL-9^−/−^ mice receiving IL-17A neutralization demonstrated significant higher *Pneumocystis* burden than IL-9^−/−^ mice injected with isotype control [(5.38 ± 1.19) × 10^8^ vs. (7.60 ± 1.95) × 10^7^ rRNA copies/lung, *P* < 0.01] (Figure 8D). The above results proved that IL-17A neutralization in IL-9^−/−^ mice could reverse the significant changes between IL-9^−/−^ and WT PCP mice. Taken together, IL-17A contributed to the lower *Pneumocystis* burden in IL-9^−/−^ infected mice.

## Discussion

*Pneumocystis* pneumonia has been a real challenge to globe physicians because of the severe conditions and high mortality it caused in immunocompromised patients. The required conditions for *Pneumocystis* culture *in vitro* remain unclear till now, making the study of its life cycle, biology and related immune response more difficult (38). Although it has been well accepted that CD4^+^ T cells play a major role in controlling *Pneumocystis* infection, the potential mechanism is not well understood how the specific Th subsets mediate immunity responses to fight against PCP. Th1, Th2 and Th17 responses have each been implicated in protective responses during infection. However, it remains unclear if any Th subset is absolutely necessary in controlling PCP (14).

*In vivo*, Th9 cells were the main source of IL-9 (17) with multifaced roles depending on specific diseases. IL-9^−/−^ mice were used to study the contribution of IL-9/Th9 function in immune responses against a respiratory fungal infection, and we found that IL-9 was highly likely to have a negative effect on clearance of *Pneumocystis* organisms in the early stage of infection.

Several studies have demonstrated that Th17/IL-17 seems beneficial to the clearance of *Pneumocystis* organisms. In our study, IL-9^−/−^ PCP mice had increased number of Th17 cells infiltrated in the lungs, as well as higher level of IL-17A production in BALF than WT PCP mice. Our results suggested IL-9 might be detrimental to controlling *Pneumocystis* infection and IL-9 deficiency lowered the *Pneumocystis* burden probably *via* the increasing Th17 cells. The IL-9^−/−^ PCP mice had increased *Pneumocystis* burden in lung while receiving IL-17A neutralization, providing the evidence to establish the association of enhanced Th17 responses and weak *Pneumocystis* burden. To our limited knowledge, this article is the first attempt to demonstrate the relationship between IL-9 and IL-17 during *Pneumocystis* infection.

Previous work has shown that differentiated Th17 cells had high expression of IL-9 receptor and could produce IL-9 after restimulation with transforming growth factor β and IL-4 (25), so that IL-9 might have an autocrine impact on Th17 cell differentiation regulation. Our presented data demonstrated that IL-9 deficiency could enhance Th17 cells response during *Pneumocystis* infection. In contrast, a study observed IL-9 neutralization followed by attenuated Th17 responses in an animal model of experimental autoimmune encephalomyelitis (25), implicating that IL-9 might contribute to inflammatory disease as a Th17-mediator cytokine. Conflicting findings from the results of this current study were mainly because of totally different disease models involving distinct complicated immune responses. However, Th9 cells reportedly played both aggravating and suppressive roles even in the same murine model of experimental autoimmune encephalomyelitis (24, 25, 39).

The results demonstrated that IL-9 deficiency promoted the differentiation of Th17 cells, and the mRNA expression of genes related to Th17 differentiation pathway were upregulated in IL-9^−/−^ PCP mice compared with WT group. To the contrary, Nowak et al. concluded that IL-9 could induce Th17 differentiation through performing Th17 differentiation trials from IL-9 receptor-deficient mice and WT mice (25). We made possible speculation as follows. The deficiency of IL-9 receptor might totally differ from IL-9 deficiency in effects on regulating CD4^+^ Th subset, meanwhile, different differentiation conditions were administrated in the two experiments. The underlying reasons require further study. In addition, STATs play critical role in controlling T cell differentiation (40). A published study on experimental autoimmune encephalomyelitis indicated that STAT1 and STAT3, but not STAT5, regulated IL-9 mediated IL-17 production in T cells (41). In another study on multiple sclerosis (42), IL-9 activated STAT1 and STAT5, which are known to inhibit Th17 polarization (43, 44), and reduced IL-17 production. In our murine model of *Pneumocystis* infection, PCR results from lung tissue showed upregulated expressions of STAT3 and STAT5 (Figure 4B) and similar levels of STAT1 expression (Figure S3 in Supplementary Material) in IL-9^−/−^ mice compared with WT mice. As STAT3 was reported to be a central component of Th17-dependent autoimmune processes (45) and could compete with STAT5 by binding to specific sites to promote the induction of Th17 cells (44), the enhanced Th17 response might result from multiple factors, and the upregulated STAT3 and interactions in STATs should be taken into consideration. On the basis of studies *in vitro* and *in vivo* of autoimmune diseases, we hope to explore the effect and interaction of STATs on Th17 response in *Pneumocystis* infection.

IL-23, mainly produced by activated antigen-presenting cells, such as macrophages and dendritic cells (46), is widely reported to play an important role in controlling Th17 cells development. Although IL-23 is not the differentiation factor for the generation of Th17 cells (47), there is increasing evidence that IL-23 promotes the survival and expansion of Th17 cells (15, 48–50). As previously reported, IL-23 was shown to inhibit the expression of IL-9 *in vitro* (24), however, little was recorded about the role of IL-9 on IL-23 expression. In our experiments, though two groups presented similar frequencies of Th17 cells expressing IL-23R in BALF by flow cytometry, IL-23R mRNA expression in lung and IL-23 concentration in BALF were elevated in IL-9^−/−^ mice during *Pneumocystis* infection contrasted with WT mice. Given the results from previous study and current experiments, it seemed that IL-9 and IL-23 could suppress the production of each other. What’s more, IL-23 knockout mice demonstrated impaired ability to clear *Pneumocystis* organism (12), it corresponded with the result that IL-9^−/−^ mice hosted lower *Pneumocystis* burden in lung with higher IL-23 concentration in BALF. It was confirmed that IL-23 expression can be induced during *Pneumocystis* infection through detecting the IL-23 concentration by ELISA in our study, indicating IL-23 played a role in host defense against *Pneumocystis* organism. However, additional studies are needed to explore the mechanism of regulation between IL-9 and IL-23 expression. Enhanced Th17 cells response was observed in IL-9^−/−^ PCP mice, and the reasons for increasing Th17 cells and upregulated IL-17 signaling pathway remained unclear. Considering the ability of IL-23 to promote and maintain Th17 development, it may be the possibility of promoting Th17 cell numbers *via* the IL-23-dependent mean, which required further confirmation.

A study in a murine model of rheumatoid arthritis revealed that Th17 cells could be drawn to sites of IL-17-driven inflammation through CCR6 detected on Th17 cells, which is the receptor for CCL20 (51). Little was known about the means by which Th17 cells infiltrated into alveolar spaces in PCP. Whether CCR6/CCL20 participates in the above process or not deserves further investigation. However, in the current study, the mechanism leading to the different driven Th17 cells count in alveolar space might differ from that in rheumatoid arthritis for the similar concentrations of CCL20 in BALF from both IL-9^−/−^ and WT PCP mice.

Sufficient evidence has been provided regarding the importance of AMs in fighting against PCP in previous studies (35, 52, 53). More AMs was observed from the BALF in IL-9^−/−^ PCP mice contrasted with WT mice, because of macrophages’ ability to produce IL-23, indicating increasing number of AMs might be the reason for higher IL-23 in BALF from IL-9^−/−^ PCP mice. Our experiment revealed that additional IL-17 could protect AMs from apoptosis *in vitro*, revealing that IL-17 might enhance the clearance of *Pneumocystis* organisms through increasing living AMs, which needs further research. In a study of Plasmodium berghei infection in mice, a striking reduction in splenic macrophages was observed in IL-17 knockout mice, which were highly susceptible to Plasmodium berghei infection, suggesting that IL-17 was important for maintenance of splenic macrophages (54). And adoptive transfer macrophages confirmed the value of IL-17 to macrophages accumulation in macrophage-depleted mice. The above data supported our results that IL-17A neutralization in IL-9^−/−^ PCP mice could significantly decrease the AM number in BALF, making it necessary to explore the relationship between IL-17 and AMs in PCP pathogenesis.

Neutrophils are known to play important role in various pulmonary infections, which could be recruited by IL-17A in the lungs (49, 55–57). Whereas it was reported that neutrophils did not have a major role in the clearance of *Pneumocystis* organisms (58), elevated pulmonary neutrophils seemed to be a risk factor for poor outcome in PCP patients (59). And compared with HIV PCP patients, non-HIV PCP patients hosted lower *Pneumocystis* burden and more neutrophils in the BALF with worse prognosis, suggesting neutrophil-related inflammation injury might impact the process of PCP. Since neutrophils did not appear to play a vital role during host fighting against *Pneumocystis* infection, it should be stated, however, the above studies do not draw the final conclusion to deny the role of neutrophils in response to *Pneumocystis* infection. In the current study, the neutrophils recruitment might serve as the indicator to present the difference of Th17/IL-17 from the two groups. The function of pulmonary neutrophils in human and murine individuals infected with *Pneumocystis* organisms is worthy further study.

Although CD4^+^ T cells and AMs are critical for resolution of *Pneumocystis*, there is evidence that B cells and *Pneumocystis*-specific antibody responses also played a role in protection against *Pneumocystis* organisms (60, 61). Our data demonstrated that IL-9^−/−^ PCP mice demonstrated similar levels of B cell number, production of total serum antibodies and anti-*Pneumocystis* serum antibodies with WT PCP mice 3-week postinfection (shown in Supplementary Materials), indicating that IL-9 deficiency might play little role in the production of serum antibodies, and the difference of *Pneumocystis* burdens between WT and IL-9^−/−^ mice 3-week postinfection was largely irrelevant to the antibodies in serum. Despite the above findings, the B cell immunity and the immunoglobulin production in PCP process remain a topic worthy of study. Our previous work revealed significant B lymphocytes suppression and B cell related pathways in corticosteroid-treated hosts with PCP by a lung microarray study (62), highlighting the role of B cell immunity in PCP.

In summary, PCP remains one of the most serious complications in immunocompromised patients, and the mechanisms related to immunity against *Pneumocystis* infection are not well understood. Although similar basic clearance of *Pneumocystis* organisms was achieved in both WT and IL-9^−/−^ PCP mice, IL-9 deficiency could lower *Pneumocystis* organism burden and promote pulmonary Th17 cells response in the early stage of infection. These findings provide evidence that IL-9 may be involved in the immunity against *Pneumocystis* infection, and IL-9 deficiency could reduce *Pneumocystis* burden *via* promoting Th17 immunity response. The current study may provide insights into the potential target use of IL-9-based therapy for PCP in future.

## Ethics Statement

All animal work was conformed to the Ethics Committee of Capital Medical University.

## Author Contributions

Z-HT conceived and designed the study. TL, H-MR, CZ, and KZ acquired, analyzed, and interpreted the information. Z-HT, TL, and KZ wrote, reviewed, and/or revised the manuscript. H-MR and CZ proofread and formatted.

## Conflict of Interest Statement

The authors declare that the research was conducted in the absence of any commercial or financial relationships that could be construed as a potential conflict of interest.
