# Supplementary material for: IL-9 Deficiency Promotes Pulmonary Th17 Response in Murine Model of Pneumocystis Infection
Source: Front Immunol. 2018 May 25;9:1118. doi: 10.3389/fimmu.2018.01118 (PMC5980981; doi:10.3389/fimmu.2018.01118)
Supplement: Supplementary file 1 [file Data_Sheet_1.PDF]

Supplementary Material

IL-9 Deficiency Promotes Pulmonary Th17 Response in Murine Model of *Pneumocystis* Infection

Ting Li, Heng-Mo Rong, Chao Zhang, Kan Zhai, Zhao-Hui Tong *

*** Correspondence:** Zhao-Hui Tong; tongzhaohuicy@sina.com

1. Supplementary Table S1

Supplementary Table S1. Primers used in real-time PCR for gene mRNA expression

Genes	Forward Primer(5’-3’)	Reverse Primer(5’-3’)
GAPDH	AGGTCGGTGTGAACGGATTTG	TGTAGACCATGTAGTTGAGGTCA
IL-17A	TTTAACTCCCTTGGCGCAAAA	CTTTCCCTCCGCATTGACAC
IL-17RA	ATGAGTGGATCTGTTGCCCTA	GAGAGAGCACAGGATTTCACAC
Traf6	AAAGCGAGAGATTCTTTCCCTG	ACTGGGGACAATTCACTAGAGC
Nfkb1	GGAGGCATGTTCGGTAGTGG	CCCTGCGTTGGATTTCGTG
Nfkbia	TGAAGGACGAGGAGTACGAGC	TTCGTGGATGATTGCCAAGTG
CXCL1	CTGGGATTCACCTCAAGAACATC	CAGGGTCAAGGCAAGCCTC

CXCL5	GTTCCATCTCGCCATTCATGC	GCGGCTATGACTGAGGAAGG
CCL20	GCCTCTCGTACATACAGACGC	CCAGTTCTGCTTTGGATCAGC
IL-6	TAGTCCTTCCTACCCCAATTTCC	TTGGTCCTTAGCCACTCCTTC
TNF-α	CAGGCGGTGCCTATGTCTC	CGATCACCCCGAAGTTCAGTAG
Csf3	ATGGCTCAACTTTCTGCCCAG	CTGACAGTGACCAGGGGAAC
Csf2	GGCCTTGGAAGCATGTAGAGG	GGAGAACTCGTTAGAGACGACTT
Lcn2	TGGCCCTGAGTGTCATGTG	CTCTTGTAGCTCATAGATGGTGC
MMP13	CTTCTTCTTGTTGAGCTGGACTC	CTGTGGAGGTCACTGTAGACT
Fosb	TTTTCCCGGAGACTACGACTC	GTGATTGCGGTGACCGTTG
RORγt	GACCCACACCTCACAAATTGA	AGTAGGCCACATTACACTGCT
RORa	GTGGAGACAAATCGTCAGGAAT	TGGTCCGATCAATCAAACAGTTC
IL-23R	TTCAGATGGGCATGAATGTTTCT	CCAAATCCGAGCTGTTGTTCTAT
STAT3	CAATACCATTGACCTGCCGAT	GAGCGACTCAAACTGCCCT

Smad2	ATGTCGTCCATCTTGCCATTC	AACCGTCCTGTTTTCTTTAGCTT
p38	TGACCCTTATGACCAGTCCTTT	GTCAGGCTCTTCCACTCATCTAT
mTOR	ACCGGCACACATTTGAAGAAG	CTCGTTGAGGATCAGCAAGG
STAT5	CGCCAGATGCAAGTGTTGTAT	TCCTGGGGATTATCCAAGTCAAT
STAT6	CTCTGTGGGGCCTAATTTCCA	CATCTGAACCGACCAGGAACT

2. Supplementary Figure 1

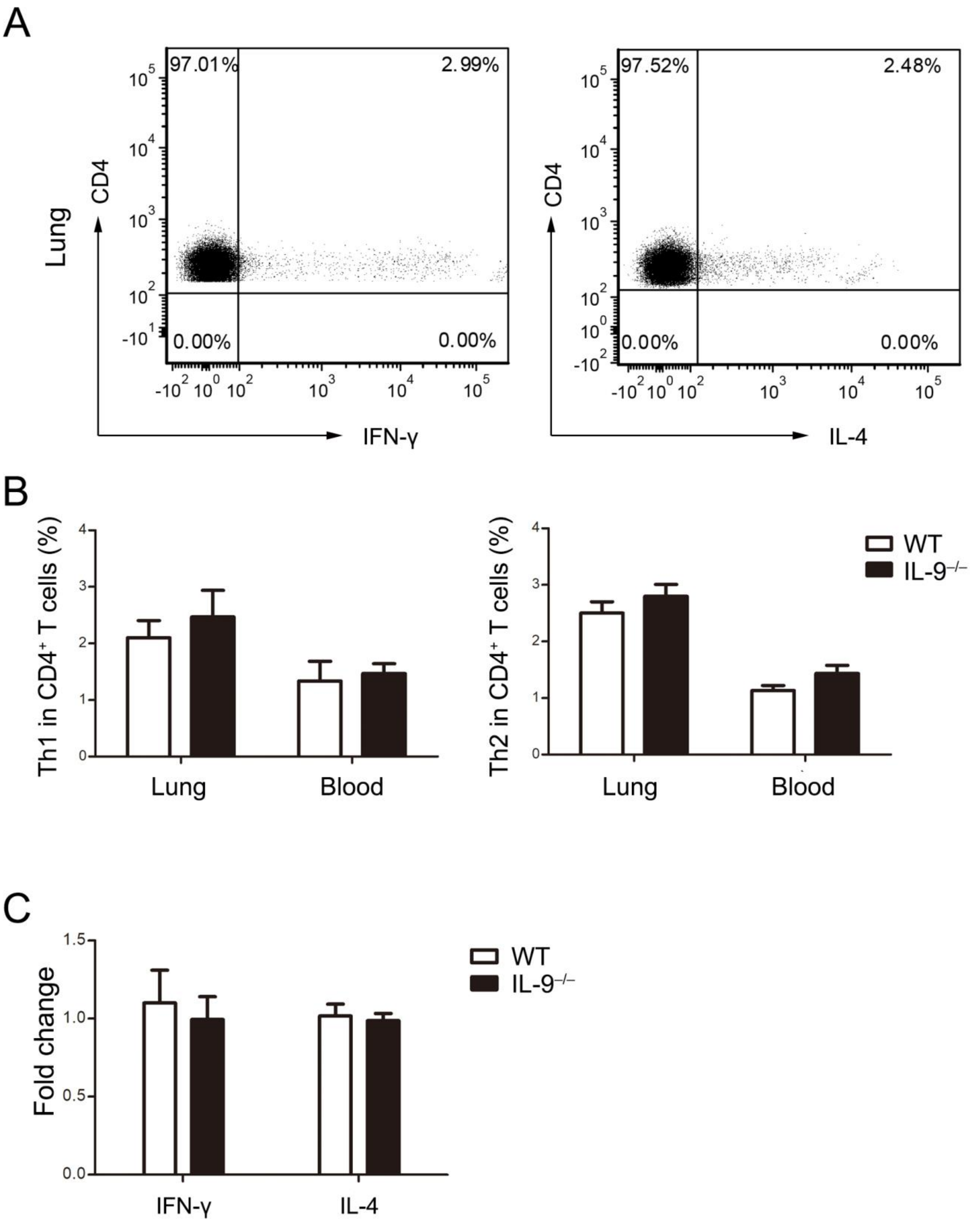

Supplementary Figure 1. Th1 and Th2 cell frequencies didn't differ in WT and IL-$9^{-/-}$ PCP mice. (A)Representative flow cytometric dot plots showed the percentage of Th1 ($CD4^{+}$IFN-γ^{+}) and Th2 ($CD4^{+}$IL-4^{+}) subset in $CD4^{+}$ T lymphocytes in lung from WT and IL-$9^{-/-}$ mice 3 week post *Pneumocystis* infection. (B) The comparisons of Th1 and Th2 percentages were shown in lung and blood between the above two groups. Data were expressed as mean ± SEM (n = 4-5 per group). (C) The lung mRNA expressions of IFN-γ and IL-4 were detected through real-time PCR and compared

between WT PCP and IL-9$^{-/-}$ PCP mice at 3 week post infection. The fold change was figured out using the $2^{-\triangle\triangle CT}$ normalized to GAPDH with 7 samples per group. The results were representative of three independent experiments. All $P > 0.05$ compared with WT group by Student's t test.

3.Supplementary Figure 2

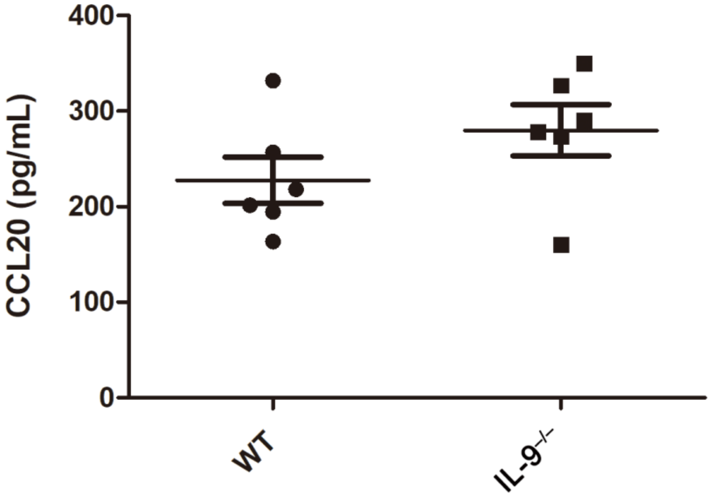

Supplementary Figure 2. IL-9$^{-/-}$ mice had similar level of CCL20 in BALF with WT mice post Pneumocystis infection. The comparison of CCL20 in BALF was shown between the above two groups. Data were expressed as mean ± SEM (n = 6 per group). The results were representative of three independent experiments. $P = 0.179$ compared with WT group by Student's t test.

4. STAT1 expression by real-time PCR in lungs from WT and IL-9$^{-/-}$ PCP mice

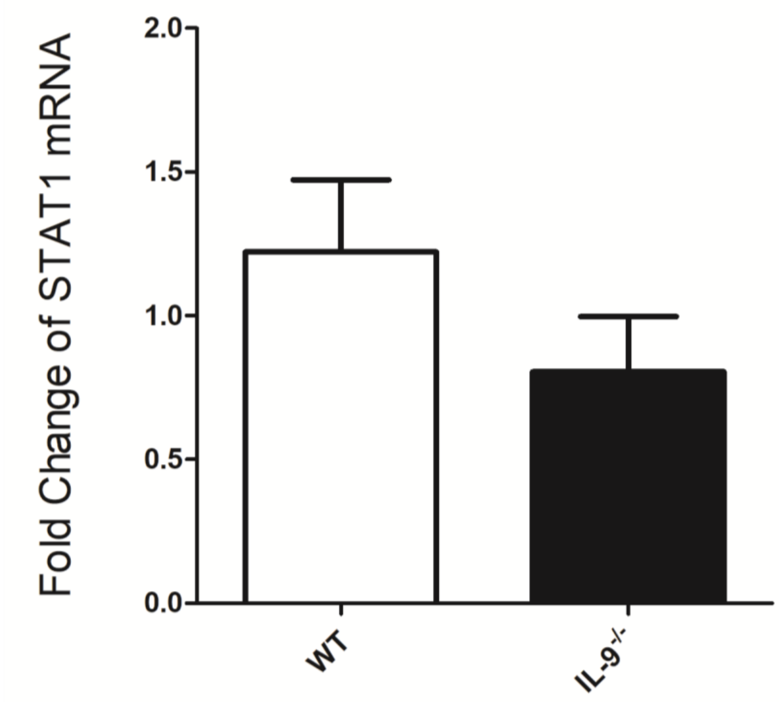

Supplementary Figure 3. STAT1 mRNA expression in lungs from WT and IL-$9^{-/-}$ PCP mice . The whole-lung RNA was extracted from WT and IL-$9^{-/-}$ mice at 3 week post-infection. STAT1 was detected through real-time PCR for mRNA expression between the two groups. The fold change was figured out using the $2^{-\Delta\Delta CT}$ normalized to GAPDH. Reactions were performed in triplicate for n = 6 samples per group. Data were presented as mean ± SEM and the graphs were representative of 3 independent experiments. P = 0.216 compared with WT group by Student’s t test.

5. Supplementary materials about the B cell count and antibodies between WT and IL-$9^{-/-}$ PCP mice

Materials and Methods

Flow cytometry

All of the different fluorochrome conjugated monoclonal antibodies (mAbs) were purchased from eBioscience (San Diego, CA), including anti-CD3, -CD19, -IgM and -IgG. The cells from blood and lung were incubated with the above mAbs for 20min at 4°C, then washed and resuspended in PBS for flow cytometric analysis (FACS Canto II; BD Biosciences, San Jose, CA).

Detection of antibodies in mouse serum

Total IgG and IgM levels in serum were measured by commercial ELISA kits (Thermo Fisher Scientific, Waltham, MA) according to the manufacturer's instructions.

Anti-*Pneumocystis* murina serum antibodies were measured by ELISA as previously described(1-4). Briefly, the *Pneumocystis* murina soluble total protein was prepared by sonication of partially purified *Pneumocystis* organisms obtained from heavily *Pneumocystis*-infected SCID mouse lungs. Flat-bottom microtiter plate (Corning, NY) was coated with the above soluble Pneumocystis antigens (10µg of protein per ml) at 37°C for 2 hours, and then coated wells were blocked with 5% milk plus 5% goat serum in PBS with 0.05% Tween 20 for 1 hour. Test serum samples were diluted 1:100 in 5% milk plus 5% goat serum in PBS with 0.05% Tween 20 and incubated in plates overnight at 4 ℃. The serum collected from normal un-infected mice was used for negative control. After washing, the wells were incubated with HPR-conjugated goat anti mouse IgG (Abcam, Cambridge, UK) diluted 1:1000 in 5% milk plus 5% goat serum in PBS with 0.05% Tween 20 for 1 hour at room temperature. After washing, 100ul TMB substrate (Thermo Fisher Scientific, Waltham, MA) was added to each well for 30 min and the stop solution was added to stop reaction. Optical density (450nm) was read within 1 hour by Thermo Scientific Varioskan Flash.

Result

No differences were found in B cell and antibody production between WT and IL-9$^{-/-}$ PCP mice 3 week post-infection

The contribution of B cells and antibodies in clearance of *Pneumocystis* organisms cannot be neglected, so it was essential to figure out whether B cells and antibodies caused the difference of *Pneumocystis* burden between WT and IL-9$^{-/-}$ mice 3 week post-infection. The proportion of B cells in lymphocytes and B cells absolute count were measured by flow cytometry. It was observed that there were no significant differences between WT and IL-9$^{-/-}$ PCP mice from lung and blood in B cell proportions and numbers [all P > 0.05](Supplement Figure 4A). Further, the IgM positive B cell proportion and IgG positive B cell proportion didn't differ in blood and lung between the two groups [all P > 0.05](Supplement Figure 4B). The serum obtained from WT and IL-9$^{-/-}$ mice 3 week post-infection were detected for IgM and IgG concentrations using ELISA, indicating that IL-9$^{-/-}$ mice generated similar serum levels total IgM and IgG with WT mice post infection[all P > 0.05](Supplement Figure 4C, left and middle panel). In consideration of the importance of *Pneumocystis*-specific antibodies during infection, anti-*Pneumocystis* IgG was detected in serum from WT and IL-9$^{-/-}$ mice 3 week post-infection. With normal uninfected WT mice as negative control, both WT PCP and IL-9$^{-/-}$ PCP mice had detectable levels of anti-*Pneumocystis* IgG. However, there were no significant differences between IL-9$^{-/-}$ and WT mice at 3 week post infection [P > 0.05] (Supplement Figure 4C, right panel). The above findings might suggest that IL-9 deficiency didn't affect the production of serum antibodies, and the difference of *Pneumocystis* burdens between WT and IL-9$^{-/-}$ mice 3 weeks post infection was largely irrelevant to the antibodies in serum.

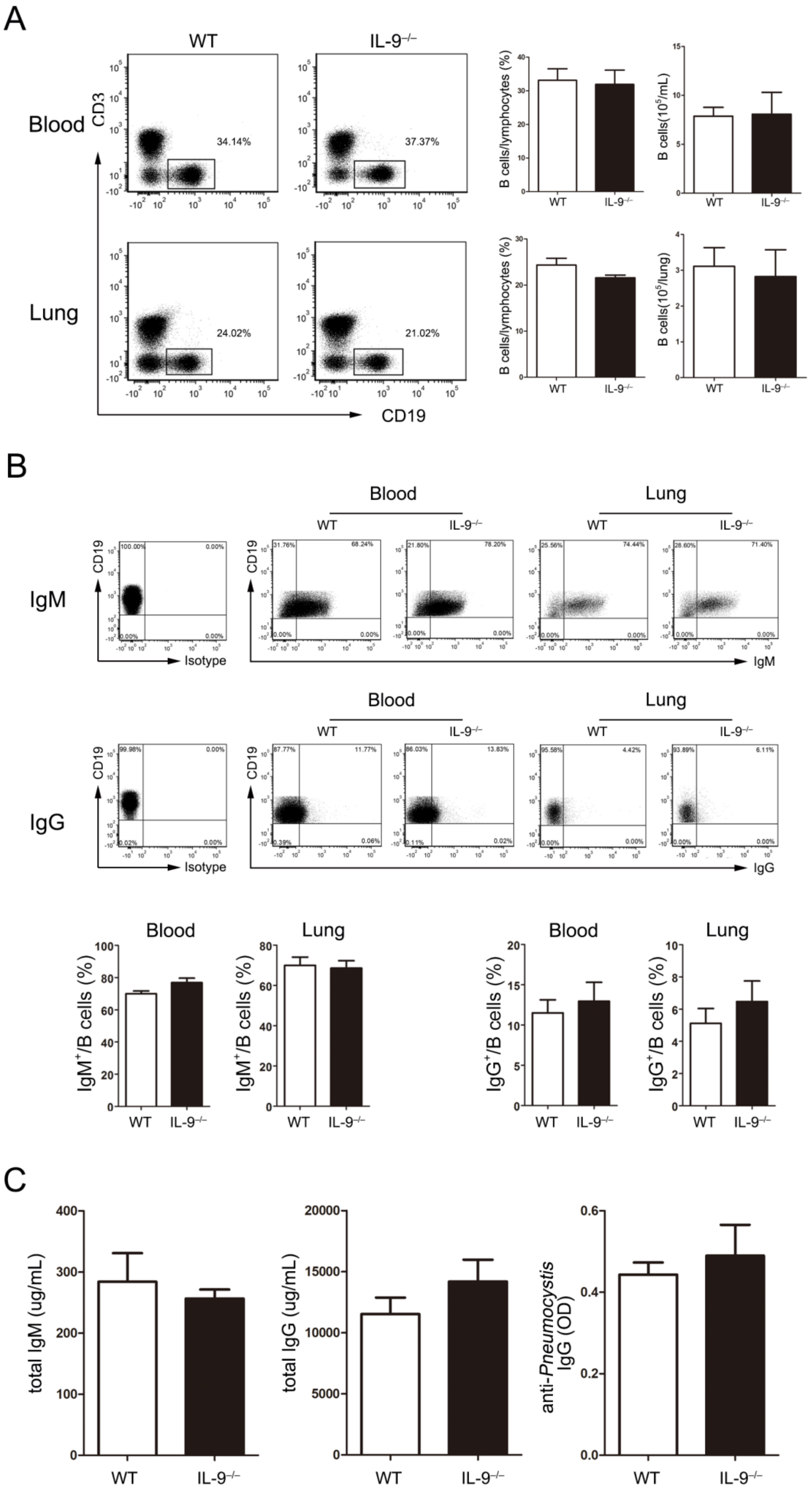
A
WT
IL-9−/−
Blood
Lung
CD3
CD19
34.14%
37.37%
24.02%
21.02%
B cells/lymphocytes (%)
B cells(10^5/mL)
B cells(10^5/lung)
B
Blood
Lung
IgM
IgG
CD19
Isotype
100.00%
99.98%
Blood
Lung
IgM+/B cells (%)
IgG+/B cells (%)
C
total IgM (ug/mL)
total IgG (ug/mL)
anti-Pneumocystis IgG (OD)

82

Supplementary Figure 4. No significant difference was found in B cell and antibody production between WT and IL-9$^{-/-}$ PCP mice 3 week post-infection. The WT and IL-9$^{-/-}$ mice were sacrificed 3 weeks post *Pneumocystis* infection. (A)Representative flow cytometric dot plots showed the percentages of $CD3^{-}CD19^{+}$ cells (B cells) in lymphocytes in blood and lung obtained from the two groups (left panel). The comparisons of B cells proportion and absolute number were shown from blood and lung in WT and IL-9$^{-/-}$ PCP mice (right panel). (B) Cells were gated from $CD3^{-}CD19^{+}$ cells (B cells), and the representative flow cytometric dot plots showed the percentages of IgM^{+} (top panel)or IgG^{+} (middle panel) cells in B cells in blood and lung obtained from the two groups. The comparisons of IgM^{+} proportion (bottom panel, left) and IgG^{+} proportion (bottom panel, right) were shown from blood and lung in WT and IL-9$^{-/-}$ PCP mice. (C) The antibody levels in the serum were detected by ELISA. The comparisons of total IgM (left panel), total IgG (middle panel), anti-*Pneumocystis* IgG (right panel) were shown in mouse serum between WT and IL-9$^{-/-}$ PCP mice. Data were presented as mean ±SEM from 4 mice per group. The results were representative of three independent experiments. All $P > 0.05$ tested by Student's t test between WT and IL-9$^{-/-}$ groups.

References

1. Harmsen AG, Chen W, Gigliotti F. Active immunity to Pneumocystis carinii reinfection in T-cell-depleted mice. *Infection and immunity* (1995) 63(7):2391-5. PubMed PMID: 7790048; PubMed Central PMCID: PMC173319.

2. Lund FE, Schuer K, Hollifield M, Randall TD, Garvy BA. Clearance of Pneumocystis carinii in mice is dependent on B cells but not on P carinii-specific antibody. *Journal of immunology* (2003) 171(3):1423-30. PubMed PMID: 12874234.

3. Bishop LR, Helman D, Kovacs JA. Discordant antibody and cellular responses to Pneumocystis major surface glycoprotein variants in mice. *BMC immunology* (2012) 13:39. doi: 10.1186/1471-2172-13-39. PubMed PMID: 22788748; PubMed Central PMCID: PMC3411419.

4. Perez-Nazario N, Rangel-Moreno J, O'Reilly MA, Pasparakis M, Gigliotti F, Wright TW. Selective ablation of lung epithelial IKK2 impairs pulmonary Th17 responses and delays the clearance of Pneumocystis. *Journal of immunology* (2013) 191(9):4720-30. doi: 10.4049/jimmunol.1301679. PubMed PMID: 24078701; PubMed Central PMCID: PMC3811920.
